# Regional inequity in complete antenatal services and public emergency obstetric care is associated with greater burden of maternal deaths: analysis from consecutive district level facility survey of Karnataka, India

**DOI:** 10.1186/s12939-017-0573-3

**Published:** 2017-05-11

**Authors:** M. Himanshu, Carina Källestål

**Affiliations:** 1 Rajiv Gandhi Institute of Public Health and Centre for Disease Control, Rajiv Gandhi University of Health Sciences, Karnataka, 26/27-1 33rd cross,18th main 4th T block Jayanagar, Bangalore, 560041 India; 2 International Maternal and Child Health, Department of Woman and Child Health, Uppsala University, Drottningatan 4, 751 85 Uppsala, Sweden

**Keywords:** Inequity, Maternal, Antenatal, Emergency, Obstetric, Mortality, District, Karnataka, India, Theil’s

## Abstract

**Background:**

This equity focused evaluation analyses change in inter-district inequity of maternal health services (MHS) in Karnataka state between 2006–07 & 2012–13, alongside association of MHS inequity with distribution of maternal deaths.

**Methods:**

Repeated cross-sectional analysis of inequity and decomposition was done on nine district level MHS indicators using Theil’s T index. Data was obtained from population linked district level facility surveys and health information systems.

**Results:**

Inequity in births attended by skill birth attendants decreased the most (83.16%) among six other MHS indicators. Community provision of comprehensive emergency obstetric care strategy remained stagnant. Districts with higher complete antenatal care share and C-sections in public settings had lesser share of state’s maternal deaths (R^2^ = 0.29, *p* = 0.004). 5 districts suffered perpetual inequity of MHS with relatively greater burden of maternal deaths.

**Conclusion:**

First 6 years of national rural health mission increased coverage of MHS and decreased regional inequity albeit non-uniformly. Distribution of system driven interventions of complete ANC and C-sections appear to determine decrease of maternal mortality in Karnataka.

**Electronic supplementary material:**

The online version of this article (doi:10.1186/s12939-017-0573-3) contains supplementary material, which is available to authorized users.

## Background

National maternal mortality ratio (MMR) estimates often mask variations between regions and sub-groups of populations. While estimated MMR of India - a low-middle income country, was 174/100,000 live births in 2015 [1], large populations lived in regions with MMR between 285 and 310/100,000 live births [2, 3]. Universal access to comprehensive reproductive health services including Emergency Obstetric Care (EmOC) remains an important determinant of low MMR [4, 5]. Inequity in access to maternal health services renders greater burden of maternal deaths [6]. In India, inter-state disparities in antenatal care (ANC) coverage, and births attended by skilled birth attendants (%SBA) has been reported [7–9]. Coverage gap and inequity in distribution of public maternal health services is strongly associated with sluggish decrease in MMR [8].

Provision of universal access to ANC, SBA and EmOC services are strategies of Indian government to reduce MMR. The Indian government provides public health policy direction and implementation framework periodically; while responsibility of execution remains with state governments. Each of India’s 29 states is divided into administrative districts having one tertiary public hospital each. Primary and secondary health centres in the district are linked to the tertiary hospital thus forming a district health system. The district administration is the lowest nodal point of health program implementation; forming a unit of monitoring and evaluation of health programs.

Since 2005, maternal health strategies in India, are implemented through the National Rural health Mission (NRHM), which targets an MMR of 100/100,000 live births by 2017 [10, 11]. NRHM, was launched in 2005 to strengthen health systems in rural areas so as to provide accessible, affordable and responsive health care. Major health programs addressing public health burden were amalgamated with NRHM. The mission provides a framework of fund flow from Indian government to the states; for health systems strengthening which includes infrastructure development, human resource augmentation, emergency response, access to products, technology and community engagement [10]. Increasing service coverage and community level provision of comprehensive EmOC (CEmOC) by up gradation of all first referral units (FRUs) are key strategies of maternal health component of NRHM [11]. Post NRHM, India recorded a steep decrease in total fertility rate and MMR with doubling of institutional deliveries [11]. Government agencies periodically reported select monitoring indicators from sample districts; providing trends in perinatal service coverage, human resource availability, commodity stocks and density of EmOC facilities [3, 12–14]. Independent researchers studied output and outcome indicators of maternal health system often in few districts and sometimes on nationally representative data [7, 9, 15–18].

Apart from monitoring secular trends, equity-focused approach is recommended to monitor and assess health-system performance [19]. Increased maternal health services sans equity perpetuate health inequality between sub-groups of women. As women in disadvantage groups suffer from inequality, MMR is bound to stagnate over time. Moreover, denial of equitable share in growth of services to sub-groups of women is unjust and violates reproductive rights. Hence studies applying equity metrics could yield a knowledge base enabling equity focused process of decision making in health care reforms [19]. Particularly so, to identify disadvantaged groups and plan targeted interventions.

Previously, studies reported interstate inequities in maternal health service coverage using nationally representative data [7, 8, 17]. However, most studies analysed inequity in dimensions of income, socioeconomic status, literacy and age while inequity across administrative regions is seldom reported [20]. Also, most studies used simple equity metrics to analyse inequity; although two studies using complex equity metrics, analysed inequity across socio-economic dimension. [8, 21]. Nevertheless, inequity of human resource distribution in maternal health system using complex equity metrics has been better described compared to coverage indicators across dimensions [22, 23]. Thus studies analysing inequity along regional dimension [inter-district inequity] using complex equity metrics are lacking.

As maternal health component of NRHM is implemented through district health system, inter-district inequity analysis of a state could provide evidence for resource allocation decisions. In India, although most districts are likely to be populated by all socio-economic groups, clustering of lower socio-economic groups in certain districts of a state is not uncommon. Thus inter-district inequity in maternal health service and MMR serve as a proxy for other dimensions of inequity in populations. Further, complex metrics, unlike simple measures, quantify inequity across all subgroups in a population, considering population size [24]. Complex equity metix could be used to monitor change in inequity over time and study health system factors associated with inequity [24]. Thus, this study aims to analyse change in inter-district inequity of maternal health service coverage and density of EmOC services within Karnataka state between 2006–07 and 2012–13. Also, the paper attempts to study associations of inequity in maternal health service coverage and MMR using maternal death data of Karnataka in 2013–14. The associations could provisionally identify predictors of regional disparity in MMR in low-income contexts

## Methods

### Context

Karnataka, a major south Indian state had population of 61.1 million in 2011 (India; 1.221 billion). 30 districts form the state which has significant regional variation in socio-economic status and health indicators. 6 of the 30 districts are constitutionally recognized under-developed regions. The State recorded MMR of 144 in 2013 in contrast to national MMR of 178 per 100,000 live births. In 2007, Karnataka recorded MMR of 178 while India recorded MMR of 212 per 100,000 live births [25, 26].

### Study design and indicators

Repeated cross-sectional analysis of inequity on maternal health coverage and facility data from population representative surveys at two time periods was conducted. Three perinatal indicators – ANC, Complete ANC, %SBA; three facility density indicators – density of Basic EmOC (BEmOC, CEmOC) facilities, % FRUs offering CEmOC and two proxy indicators of CEmOC - proportion of all deliveries through Caesarean section (C-Section) [27] formed maternal health service coverage indicators. MMR due to direct obstetric causes (MMR-DOC) was the outcome indicator for decomposition analysis. MMR-DOC was used as proxy indicator for regional MMR Table 1 summarizes definitions of maternal health service coverage indicators used in this study.

**Table 1:** Definitions of maternal health coverage indicators used

Sl no.	Indicator	Definition
1.	Antenatal care	Percentage of pregnant women who received any antenatal check-up in a district among all estimated pregnancies in the district during the survey period [25]
2.	Complete antenatal care	Percentage of pregnant women who had at least three visits for antenatal check-up, one tetanus injection received and 100 iron and folic acid tablets or adequate amount of syrup consumed in a district among all estimated pregnancies in the district during the survey period [25].
3.	Percentage delivery attended by skilled birth attendants	Percentage of deliveries that were attended by a skilled health personal in a district among all deliveries occurring in a district during the survey period [25]
4.	Basic Emergency Obstetric Care	A facility that provides six basic signal functions of obstetric emergency (parenteral antibiotics, uterotonic durgs, parenteral anticonvulsants, manual removal of placenta, assisted vaginal delivery and neonatal resuscitation) defined by the World Health Organization [24].
5.	Comprehensive Emergency Obstetric Care	A facility that provides six basic signal functions of obstetric emergency along with caesarean section and blood transfusion [24].
6.	First referral unit	A comprehensive emergency obstetric care facility, most proximate to a primary health centre [11]
7.	Percentage Caesarean sections	Percentage of deliveries conducted by caesarean section in a district among all deliveries in the district during the survey period [25]

State and district wise data of coverage and facility indicators were retrieved from third and fourth rounds (fact sheets) of District Level Household and Facility Surveys [DLHS]; conducted in 2006–07 and 2012–13 respectively [25, 28]. DLHS are population linked multistage stratified surveys of reproductive, child health services and health care facilities. Survey methods of DLHS are described elsewhere [28, 29]. While data of DLHS-3 are available in public domain, only district level fact sheets of DLHS-4 were available at the time of analysis. This difference in data source did not affect analysis as data in fact sheets were adjusted for population weights.

Perinatal indicators were available as proportion of service covered among women who had live/still births in the survey period[s]. Total BEmOC facilities were extrapolated from representative proportion of PHCs in a district providing BEmOC services. However, other maternal health facility indicators were available as total numbers. Further, density of BEmOC and CEmOC facilities per 500,000 people was calculated using population data from census 2001 and 2011 [30]. Census data was also used to calculate population weights for computing Theil’s T index, as described later. All district and state level estimates for study indicators considered were adjusted for respective population weights [28].

Average state and district MMR-DOC were calculated from maternal death data of Health Management and Informatics System, Government of India [31], using the following formula;

$$ \frac{\begin{array}{c}\hfill \mathrm{MMR}\hbox{-} \mathrm{DOC} = \mathrm{Total}\ \mathrm{Maternal}\ \mathrm{deaths}\ \mathrm{in}\ \mathrm{a}\ \mathrm{district}\ /\mathrm{state}\ \mathrm{due}\ \mathrm{to}\ \mathrm{direct}\ \mathrm{obstetric}\ \mathrm{causes}\ \mathrm{between}\hfill \\ {}\hfill \mathrm{April}\ 1,\ 2013\ \mathrm{a}\mathrm{nd}\ \mathrm{March}\ 31,\ 2014\ \mathrm{X}\ 100,000\hfill \end{array}}{\mathrm{Total}\ \mathrm{live}\ \mathrm{births}\ \mathrm{in}\ \mathrm{the}\ \mathrm{district}/\mathrm{state}\ \mathrm{between}\ \mathrm{April}\ 1,\ 2013\ \mathrm{a}\mathrm{nd}\ \mathrm{March}\ 31,\ 2014} $$

MMR-DOC was calculated only for year 2013–14 as maternal death data for period 2006–07 was unavailable.

### Equity metrics

Theil’s ‘T’ index was used to measure inter district inequity. For each district, Theil’s Component [TC] was calculated for all indicators at two time periods using the formula [24];

$$ {\mathrm{TC}}_{\mathrm{D}} = {\mathrm{p}}_{\mathrm{D}}\mathrm{x}\ {\mathrm{r}}_{\mathrm{D}}\mathrm{x}\  \ln \left[{\mathrm{r}}_{\mathrm{D}}\right]\ \mathrm{x}\ 1000 $$

Where for a district ‘D’, p_D_ was proportion of state population, r_D_ was ratio of the district indicator to the state indicator and Ln was natural log. TC_D_ is elastic to district’s share in the state total coverage/density. Further, Theil’s ‘T’ index was calculated for all indicators in both time periods by formula T = Σ TC [24]. Likewise, TC and Theil’s T was calculated for MMR-DOC in 2013-14. When compared between time periods, higher Theil’s ‘T’ indicated higher inequity [24]. Theil’s index was calculated using Microsoft Excel 14.0.

### Descriptive statistics

State average of coverage and facility indicators was described by percentage and density per 500,000 people respectively. Interquartile range described variance between districts. Percentage difference in Theil T from 2006–07 to 2012–13 indicated change in inter-district inequity in all indicators. Further, negative TC values of indicators at both time periods were used to identify districts at perpetual disadvantage relative to others. Also, MMR-DOC of districts with positive MMR-DOC-TC was calculated to identify excess burden of MMR-DOC associated with relative inequity.

### Decomposition analysis

Inter-district inequity of MMR-DOC was decomposed with step-wise linear regression method; to identify best fitted model(s). Initially, coverage and facility indicators in 2012–13 - correlating independently with MMR-DOC inequity were identified using Pearson correlation test. Further, indicators that significantly correlated (*p* < 0.05) with MMR-DOC-TC were used as predictors in step-wise regression analysis. Statistical analysis was performed using the software, Statistical Package for Social Sciences 20.0, IBM corporation.

### Ethics statement

This study was based on data from population surveys and health information systems provided in public domain by agencies of Government of India. Data sets contained no identity of survey participants or informants. Hence the study did not merit review by an ethical committee.

## Results

### Descriptive statistics

Karnataka state had 27 and 30 districts during DLHS-3 (2006–07) and DLHS-4 (2012–13) respectively. Increase in districts did not affect the analysis as new districts were part of other districts in 2006–07, thus contributing to district Theil’s T proportionate to their population. In 2001, state population was 52.773 million; increasing 16.61% to 63.241 million in 2011. Likewise, household sample size increased 1.62 times and that of villages increased 1.11 times between the two surveys. In 2014, 1,539 estimated maternal deaths occurred of estimated 1.273 million pregnancies [25]. In 2012–13, 94.5% of pregnant women in Karnataka sought ANC care (national average unavailable) marginally more than 90.2% in 2006–07 (national average 75.2%) [25].

Between the two surveys, state ANC and % SBA coverage increased (5% and 20.6% respectively) while that of complete ANC decreased (-4.9%). However, more than one-third districts recorded coverage below that of state average (Table 2). Likewise, state density of BEmOC and CEmOC facilities marginally increased (2.34% and 1.27% respectively). In 2012–13, 10%, 3%, and 1% of the districts had BEmOC, CEmOC and total EmOC density below recommended levels, respectively [27].

**Table 2:** State maternal service coverage and EmOC facility density in 2012-13 and 2006-07

	State average		Inter-district range (interquartile range)		Districts below state average (%)	
	2012-13	2006-07	2012-13	2006-07	2012-13	2006-07
ANC (%)	86.3	81.3	72.2 - 98.4 (14.02)	55.7 - 98.1 (18.2)	36.67	37.00
CANC (%)	46	51.1	18.7 - 74.4 (22.15)	16.7 - 91.8 (45.2)	46.67	44.40
SBA (%)	92.2	71.6	80.8 - 99.3 (7.2)	37.3 - 96.4 (16.7)	30.00	37.00
%Districts below recommend density^a^						
PHC - BEmOC density	9.82	7.48	1.51 - 20.78 (5.44)	0.00 - 17.63 (5.23)	10.00	14.80
All BEmOC density^b^	9.85	8.6	1.6 - 21.1 (7.08)	1.3 - 18.4 (5.95)	10.00	11.00
CEmOC density	1.88	0.61	0.75 - 4.66 (0.84)	0.00 - 2.05 (0.7)	3.00	85.00
FRUs with CEmOC facility (%)	23.1	22.5	0.00 – 100 (28.4)	0.0 - 71.4 (33.2)	90.00	100
Total EmOC density	11.54	9.03	2.3 - 23.4 (6.7)	2.3 - 18.9 (5.6)	10.00	11.00
%Districts below recommend fraction^c^						
C- Section Public (%)^d^	8.3	6	1 - 26.5 (7.2)	0.5 -15.5 (4.92)	33.3	48
C-Section Private (%) ^d^	14.5	8.6	8.4 -28.4 (5.8)	2.6 - 22.3 (4.15)	0	11
MMR-DOC (2013-14) per 100,000 live births	28.06	-	4.86 - 64.47 (20.87)	-	-	-

Density is per 500,000 population. ^a^– four BEmOC facilities per 500,000 population and one CEmOC facilitie per 500,000 population are recommended EmOC densities [27]; 100% of FRUs are to provide CEmOC services [11]. ^b^ – Includes PHCs and FRUs that provided BEmOC services; ^c^ – 5% is minimum recommended fraction of C-section deliveries among all deliveries in a survey period [27]; ^d^ – fraction of all deliveries conducted in public and private settings in the survey year. *ANC* Antenatal coverage, *CANC* Complete Antenatal Coverage, *SBA* Percentage of births attended by Skilled Birth Attendants, *PHC* Primary Health Centre, *BEmOC* Basic Emergency Obstetric Care, *CEmOC* Comprehensive Emergency Obstetric Care, *FRU* First Referral Unit, *C-Section* Caesarean Section, *MMR-DOC*, Maternal mortality due to Direct Obstetric Causes

In 2012–13, 23.1% of state’s FRUs provided CEmOC services which increased 0.6% from 2006-07. In 2012–13, while 100% of FRUs in two districts were CEmOC centres, 53% (16 districts) had no FRUs providing CEmOC services (Additional file 1: Table S2). Rate of C-section deliveries increased by 2.3% in public settings and 5.9% in private settings between two surveys. State MMR-DOC was 28.06 per 100,000 live births in 2013–14, with interquartile range of 20.87. 50% (15 districts) of districts recorded MMR-DOC higher than that of state average, with maximum district MMR-DOC of 64.47 per 100,000 live births.

### Test for inequity

Between 2006–07 and 2012–13, inter-district inequity, decreased in ANC, complete ANC and % SBA with largest decrease observed in %SBA coverage (Table 2). Nevertheless, considerable inter-district inequality (T = 84.5) in complete ANC continued to exist in 2012–13 (Table 3). Likewise, inter-district inequity in PHC-BEmOC and CEmoC facility density reduced by half. However, inter-district inequity in density of all BEmoC facilities and % of FRUs with CEmoC services increased. Inter-district inequity in total EmOC facilities remained unchanged (Table 2). This observation may be attributed to increase in inequity of %FRUs providing BEmOC and CEmoC services. Also, inter-district inequity in C-section delivery rate, decreased with inequity decreasing over two times in private settings over public settings (Table 3).

**Table 3:** Change in within-state (inter-district) inequity of service coverage and EmOC density between 2006-07 and 2012-13

Sl No.	Indicator	Theil T		% Change^a^
		2012-13	2006-07	
Maternal health service indicators				
1.	% Births attended by skilled birth attendants.	16.91	99.88	- 83.18
2.	Complete antenatal care coverage	194.65	278.95	- 30.22
3.	Antenatal coverage	37.29	51.17	- 27.12
Maternal health facility indicators				
4.	Density of CEmoC services	94.12	279.72	- 66.34
5.	Density of PHC with BEmOC services	105.23	208.36	- 49.49
6.	Density of all BEmOC facilities^a^	170.14	158.23	7.52
7.	% of FRUs with CEmoC services	574.40	486.84	17.98
Proxy indicators of CEmoC facilities				
8.	C-Section rate in private settings	169.56	395.64	- 57.90
9.	C-Section rate in public settings	121.46	163.98	- 25.93
Outcome indicator				
10.	Maternal Mortality ratio due to Direct Obstetric Complications	129.91	-	-

Indicators listed in descending order of change in inequity. ^a^ Includes PHCs and FRUs providing BEmoC services. ^b^ - Difference in Theil T between 2006–07 and 2012–13. PHC - Primary Health Centre; BEmOC – Basic Emergency Obstetric Care; CEmOC – Comprehensive Emergency Obstetric Care; FRU – First Referral Unit; C-Section – Caesarean Section

**Fig. 1:**
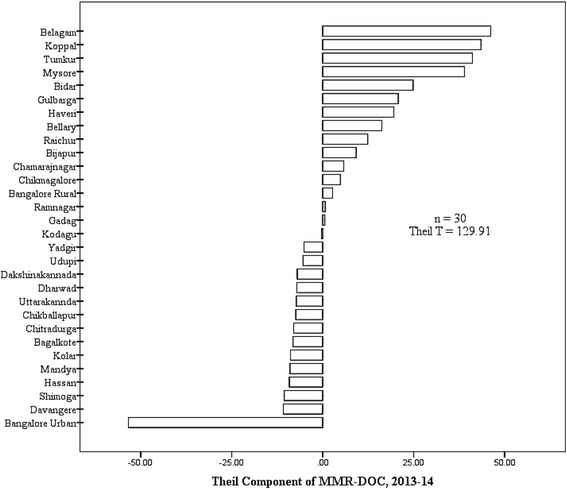
District wise Theil component of maternal mortality due to direct obstetric causes in 2013-14; Karnataka. Legend: Positive Theil component indicate higher burden of MMR-DOC relative to state average; MMR-DOC – Maternal Mortality Ratio due to Direct Obstetric Causes

The outcome indicator of MMR-DOC had Theil’s T of 129.91, signifying considerable inequity. Moreover, 50% (15 districts) had positive TC (Fig. 1). As a lower MMR-DOC is favourable, greater district TC meant greater burden of maternal deaths in the district. Mean MMR-DOC of districts with positive TC was 2.53 times more than that of districts with negative TC. Thus distribution of maternal deaths between districts was asymmetrical around the state mean; signifying underlying inequality between two sets of districts. Hypothetically, if mean MMR-DOC of districts with positive TC was equal to that of districts with negative TC, then state MMR-DOC would be 15.87 per 100,000 live births as against the actual of 28.06 per 100,000 live births (43.4% gap).

### Decomposition analysis

Districts with larger share of states ANC, complete ANC and % SBA coverage contributed lesser to state MMR-DOC as the indicators negatively correlated with MMR-DOC-TC (r = -0.43, *p* = 0.05; r = -0.45, *p* = 0.011, r = -0.40, *p* = 0.03 respectively). Among the facility indicators, density of BEmOC-TC positively correlated with MMR-DOC (r = 0.38, *p* = 0.038) while % C section rate-TC negatively correlated with MMR-DOC-TC (r = -0.4, *p* = 0.031; r = -0.4, *p* = 0.025 respectively). No other facility variables correlated with MMR-DOC-TC.

Variables that co-related with MMR-DOC-TC were tested as independent variables in a liner regression model with MMR-DOC-TC as outcome variable. The model was not significant (*p* = 0.057) and had co-linearity issues. However, in a step-wise liner regression model, predictors complete ANC-TC and % C-Section in public settings –TC were significantly (adjusted R^2^ = 0.292, *p* = 0.004) associated with MMR-DOC-TC. Further, the predictor variables were also independently associated with MMR-DOC-TC (Table 4).

**Table 4:** Association of within state inequities in service coverage (2012–13) with direct cause maternal mortality ratio (2014)

	Unadjusted model		Adjusted model^a^	
Predictor variable	Estimates	*p* value	Estimates	*p* value
*Model (R* ^*2*^ *= 0.292, p = 0.004)*				
CANC- TC	-0.45	0.011	-0.432	0.010
C-Section rate - public settings -TC	-0.40	0.031	-0.365	0.027

Estimates are standardized beta values derived from liner regression model. ^a^ Adjusted for predictors in the model. R^2^ values are adjusted for number of predictors

Five districts were on the side of disadvantage in coverage of ANC, complete ANC and %SBA in 2006–07 and 2012–13. Four of the districts (Additional file 1: Table S2) had relatively higher MMR-DOC than others in 2014 (>1 standard deviation of state average). One district had relatively lesser share of EmOC density in 2006-07 and 2012–13.

## Discussion

This equity evaluation of maternal health services in Karnataka analyses inequity change in coverage, facility and impact indicators along regional dimension over time. Further, the study analyses cross sectional associations of inter-district distribution of maternal health services with maternal deaths in 2014.

In six years, between 2006–07 and 2012–13, coverage of ANC, %SBA, density of BEmOC, CEmOC facilities and proportions of C-section deliveries increased with concomitant decrease in inter-district inequity. However, complete ANC coverage decreased; rather uniformly, as evidenced by concomitant decrease in inter-district inequity. In contrast, inequity in %FRUs providing CEmOC services increased with marginal increase (0.6%) in their number.

In 2014, inter-district variation in maternal deaths was considerable; with half of the districts contributing 2.5 times more than other half to the state MMR due to direct obstetric causes. Meanwhile, districts with higher share of state complete ANC coverage and % C-sections in public settings contributed lesser to state MMR-DOC. Furthermore, five districts suffered from perpetual inequity of maternal health service coverage and with four of the districts having relatively higher burden of maternal deaths.

Complex equity metrics of Theil’s ‘T’ index, based on population representative – weights adjusted data is central to this analysis of inequity. Theil’s index estimates inequity across different levels of aggregation and allows identification of sub-group contribution [population size adjusted] to group average [24]. Thus the index provides robust measure of regional inequity where resources are distributed across different strata of populations [32]. The index is scale invariant and flexible for use on survey data involving various health outcomes [33]. Theil’s index can be decomposed across sub-groups of populations to test relationship with health system indicators [32]. The index is valuable when measuring health system utility with marginal returns [33] such as association of perinatal services and maternal deaths.

However, this analysis does not account for inequity along other dimensions, which could exist even within advantaged districts. Further, input data is not without infirmities. While DLHS data may suffer from sampling and reporting errors, health management and information system data may suffer from reporting bias as the data reporting is not designed for research. Further, survey data of Bangalore city was obtained from municipal website [34]. Also, as municipal area of Bangalore city was different during two surveys, comparative estimates for Bangalore Urban district may be imprecise. Dakshina Kannada district records zero BEmOC centres in the sample surveyed [35]. Perhaphs, BEmOC facilities were not part of the sample. Further, all sub-divisional hospitals in a district were considered as CEmOC facility as data on CEmOC services in these hospitals was unavailable. Indian public health standards mandate these hospitals to provide CEmOC services [36]. However, a survey in 3 northern districts of Karnataka has shown otherwise [18]. Thus marginal over-estimation of CEmOC density is a possibility. Moreover, sensitivity analysis showed high elasticity of MMR-DOC to maternal death data and of regression estimates to coverage data. Hence regression estimates are valid only for the year 2014. The estimates however are representative of associations. Importantly, associations with MMR-DOC do not imply causation due to cross-sectional study design. Also, predictors of MMR-DOC analysed are non-comprehensive and reported associations may be marginally elastic to MMR in a larger context. Nevertheless, when interpreted along with limitations, results of this study contribute to evidence for programmatic and policy intervention. Tellingly, relationship of EmOC density distribution with MMR has seldom been studied in low-income settings. Evidence for EmOC strategies is largely contributed by studies from high-income settings except for Sri Lanka and Malaysia [5]. Hence, this study contributes to growing body of knowledge in system pathways of preventing maternal deaths in low income settings

Base line survey of this study (DLHS-3) was conducted in 2006–07; one year after launch of NRHM [29]. Thus, study results could reflect equity based performance of NRHM’s maternal health component in Karnataka state over 6 year period. NRHM envisages reduction in regional health disparities by increased access to quality and affordable health care [10]. A 2013 state-commissioned process evaluation report of NRHM, descriptively noted regional disparities in maternal health facilities across Karnataka, alongside need of a plan to tackle the same [14]. However, this study demonstrates reduction in inter-district inequity of perinatal services and EmOC facilities, 6 years after launch of NRHM, with plausible increase in access. A national sample evaluation also reported increase in maternal health care services and utilization across seven states post NRHM, although change in regional inequity was not evaluated [12].

**Fig. 2:**
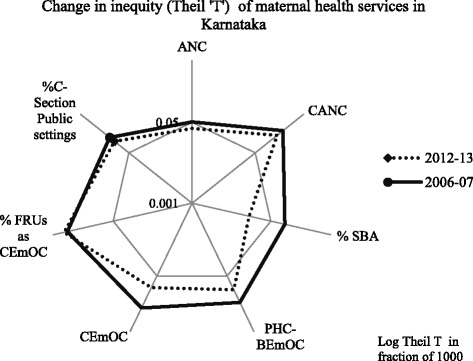
Theil ‘T’ of perinatal and EmOC service indicators in two time periods; Karnataka state. Legend: Theil T is plotted in log scale. Centre of the plot is point of equity. ANC – Antenatal coverage; CANC – Complete Antenatal Coverage; SBA – Percentage of births attended by Skilled Birth Attendants. PHC – Primary Health Centre; BEmOC – Basic Emergency Obstetric Care; CEmOC – Comprehensive Emergency Obstetric Care; FRU – First Referral Unit; C-Section – Caesarean Section

Nevertheless, inter-district inequity decrease was not uniform across services in Karnataka (Fig. 2) despite NRHM being a comprehensive health systems strengthening program. Developed economies and countries like Malaysia and Srilanka lowered MMR by community level provision of all components of maternal health care [5]. Perhaps, resource inputs were uneven or prioritised towards selected services. Systems driven interventions like complete ANC and CEmOC services are far from equity. However, ANC and %SBA have progressed near universal coverage. Incidentally, these interventions involve incentivised community health workers. Value of task shifting and community health workers in reducing maternal health service inequity is worth investigating.

Provision of universal access to ANC services and SBA are key interventions of NRHM to reduce maternal mortality [11]. Six years of NRHM intervention benefited previously under-served districts, as increased coverage in ANC services (except for complete ANC) and %SBA paralleled decrease in inter district inequity. Similarly, Ranidev et al, showed an increase in %SBA coverage and marginal decrease in inequity across socio-economic status, post NRHM in nine high focus states [21]. Studies prior to NRHM showed inequities in ANC and %SBA coverage across states and socio-economic sub-groups [8]. Thus elite capture of increased coverage in previously underserved districts cannot be ruled out.

As large proportions of maternal deaths are attributed to direct obstetric causes, universal access to EmOC facilities is advocated to reduce MMR [37]. Increased density concomitant to decreased inequity of EmOC facilities suggests service of new facilities in time between two surveys; more so in underserved areas. Also, PHCs and community health centres were upgraded to BEmOC and CEmOC centres respectively [25]. Likewise, in nine other states, EmOC densities increased with patterns similar to Karnataka state. But, trend in inequity change is unknown, after implementation of NRHM [12, 21].

Ensuring universal access of EmOC to delivering mothers requires community level provision of EmOC services [38]. Geographical access to BEmOC and CEmOC facilities are pivotal in managing potentially fatal obstetric emergencies [27]. Although envisaged by NRHM, such provision has seldom progressed except in urban/semi-urban areas. In 2012-13, 10% of districts continued to have BEmOC density below recommended levels. Meanwhile, the other NRHM strategy of upgrading all FRUs to CEmOC facilities remains stagnant in Karnataka. In a referral chain, CEmOC-FRUs are closer to a PHC, than district a hospital [11]. While about a quarter of all FRUs function as CEmOC facilities with regional concentration, no change was observed between the surveys. Mony et al, in a primary survey from select districts of north Karnataka also showed that no CEmoC facilities were available below the sub-divisional level [18]. A 2011 evaluation from seven states reported similar situation in Uttar Pradesh and Madhya Pradesh [39]. NRHM strategy of FRU up gradation warrants serious attention in Karnataka and perhaps in other states as well. Historically, MMR rapidly reduced in Britain and United states after increase in density of CEmOC services to delivering women. Similar trends have also been noted in Sri Lanka [5]. Reduction of MMR, from medium and low levels tends to depend on increased access to CEmOC services [5, 37]. Hence, adequacy of BEmOC facilities and percolation of CEmOC services to community level may be vital for Karnataka and other states to avoid MMR stagnation.

Presence of EmOC facilities may not reflect quality of service delivered. Hence, this study analysed proportion of all births by C-section which indicates access and quality of EmOC facilities. An epidemic of C-sections in private sector, plausibly accessed by upper socio-economic status women is apparent in Karnataka. Similar patterns were in high focus states as well [39]. High rates of C-sections could have negative consequences on maternal health through biological and social mechanisms [27]. However, access to C-sections in public settings remains inequitable as one-third of districts recorded below recommend rate in 2012–13. Considered together with association of %C-Sections inequity with MMR-DOC, burden of maternal deaths in one-third of districts could be avoided by equitable distribution of quality EmOC facilities. Furthermore, decrease in CEmOC facility inequity has not paralleled increased access, reflecting upon quality of facilities. Perhaps, newer EmOC facilities suffer from lack of human resources, commodities and funding [23].

Priority of EmOC over ANC and SBA coverage in low income settings has been discussed in literature [5]. Sporadic evidence from low-income settings indicated effectiveness of EmOC in preventing maternal deaths when SBA was absent [5, 40]. Nevertheless, antenatal services and CEmOC facilities appear to determine reduction of MMR in Karnataka. In a step wise regression analysis, distribution of complete ANC and C-Sections in public settings was associated with district contribution to state MMR-DOC. Perhaps, districts with mature health systems had lower MMR burden; as greater coverage of complete ANC and C-Sections in public settings are resource intensive and sensitive to systems approach. Moreover, the likelihood of maternal death prevention is higher with complete ANC than any ANC. Also, in high income settings, CEmOC density was associated with greater reduction in MMR than SBA [5]. Nevertheless, with coverage of ANC and %SBA being near universal in the state, equitable increase in complete ANC coverage and CEmOC density may hereon reduce MMR in Karnataka. Nevertheless margin of MMR reduction is speculative owing to cross-section design of this study. Also, regression of MMR inequity was non-comprehensive as indicated by small R^2^ value. The model included only health system predictors – in line with study objective; and not demographic and social indicators.

Associations observed in this study may be similar in other states of India as well. Karnataka is an economically forward state although six districts are constitutionally recognized under-developed areas [26]. Thus the state has varied socio-economic contexts including those similar to low income settings in India and abroad. Given that NRHM implementation model is same across India, patterns of maternal health services and MMR reduction may be similar. Also, a nationally representative study showed that although 82% of maternal deaths were due to direct obstetric causes, one-third of the deaths happened before onset of labour [17]. In this background, results of this study underscore equal importance of complete ANC and CEmOC services in maternal health programs of India.

Focus on sub-national health inequalities and inequity in service distribution has been minimal in contrast to achieving targeted national/state averages [41]. Inter-district inequity in maternal health services renders unacceptable maternal deaths in sub-groups of populations thus violating human rights and stagnating state MMR. District level inequity coupled with inequities in other dimensions precipitates higher burden of maternal deaths in deprived populations, thus perpetuating inequality. Focus on comprehensive maternal health service provision in perpetually disadvantaged districts is imperative. Nevertheless, NRHM appears to have reduced inter-district inequity in maternal health services with concomitant reduction in maternal deaths. Further reduction of MMR appears tougher; for it requires augmentation and fair distribution of resource intensive and systems driven services of complete ANC and CEmOC. Moreover, reduction in inequity across services has been uneven (Fig. 2); implications of which could impede achieving MMR target.

## Conclusion

In this equity-based evaluation of maternal health services, increased coverage and EmOC facility density was concomitant to decrease in inter-district inequity in Karnataka. This analysis of 2006-07 and 2012-13, coincided with first six years implementation of NRHM. Thus, decrease in regional inequity may be attributed to the NRHM model. However, complete ANC decreased uniformly across the state in the same period. Also, strategy of FRU up-gradation so as to provide CEmOC services remains stagnant. Thus, community level provision of CEmOC services is sparse with marked regional concentration. With near universal coverage of ANC and SBA, distribution and coverage of complete ANC and CEmOC services seem vital to prevent maternal deaths. This study also identified districts perpetually disadvantaged with state’s share of maternal health services. Focus on increasing ANC, SBA and complete ANC in disadvantaged districts could reduce state MMR burden; for these districts bear greater burden of state maternal deaths. This apart, state-wide increased resource allocation and systems approach to increase complete ANC coverage and community provision of CEmOC services could determine further reduction of MMR in Karnataka and similar contexts.

## Additional file

Additional file 1: Supplementary data: List of perpetually disadvantaged districts in maternal health service coverage and districts with no FRUs providing CEMOC services. (DOCX 13 kb)

## Abbreviations

ANC: Antenatal care
BEmOC: Basic emergency obstetric care
CEmOC: Comprehensive emergency obstetric care
C-Section: Caesarean section
DLHS: District level household and facility surveys
EmOC: Emergency obstetric care
FRUs: First referral units
MMR: Maternal mortality ratio
MMR-DOC: Maternal mortality ratio due to direct obstetric causes
NRHM: National rural health mission
PHC: Primary health centre
SBA: Skilled birth attendants
TC: Theil component
