# Supplementary material for: Regional inequity in complete antenatal services and public emergency obstetric care is associated with greater burden of maternal deaths: analysis from consecutive district level facility survey of Karnataka, India
Source: Int J Equity Health. 2017 May 11;16:75. doi: 10.1186/s12939-017-0573-3 (PMC5426006; doi:10.1186/s12939-017-0573-3)
Supplement: Additional file 1: — Supplementary data: List of perpetually disadvantaged districts in maternal health service coverage and districts with no FRUs providing CEMOC services. (DOCX 13 kb) [file 12939_2017_573_MOESM1_ESM.docx]

**Supplementary data to article;**

**Regional inequity in complete antenatal services and public emergency obstetric care is associated with greater burden of maternal deaths: Analysis from consecutive district level facility survey of Karnataka, India.**

In 2012-13, 23.1% of state’s FRUs provided CEmOC services which increased 0.6% from 2006-07. In 2012-13, while 100% of FRUs in two districts were CEmOC centres, 53% (16 districts) had no FRUs providing CEmOC services. Upgradation of FRUs so as to provide CEmOC services is a strategy of national rural health mission to reduce maternal mortality rate.

Table S1. Districts with no FRUs providing CEmOC facilities in 2012-13

| **SL no.** | **DISTRICT** | **SL no.** | **DISTRICT** |
| --- | --- | --- | --- |
| 1. | Bangalore rural | 9. | Gadag |
| 2. | Bangalore Urban | 10. | Kolar |
| 3. | Bellary | 11. | Raichur |
| 4. | Bidar | 12 | Ramnagar |
| 5. | Chikballapur | 13. | Shivamogga^#^ |
| 6. | Chitradurga | 14. | Udupi |
| 7. | Davangere | 15. | Uttarakannada |
| 8. | Dharwad^#^ | 16. | Yadgir |

# - The survey recorded as 'No information available'. FRU – First Referral Unit

Negative Theil component values of indicators at both time periods were used to identify districts at perpetual disadvantage relative to others. Also, MMR-DOC of districts with positive MMR-DOC-TC was calculated to identify excess burden of MMR-DOC associated with relative inequity.

Table S2. Disadvantaged districts due to relative inequity in maternal service coverage and EmOC facility density in 2012-13 and 2006-07

| **Sl no.** | **ANC%, CANC% and Birth by SBA%** | **Density of BEmoC and CEmoC facilities** |
| --- | --- | --- |
| 1 | Bellary | Dharwad |
| 2 | Bijapur |  |
| 3 | Koppala |  |
| 4 | Raichur |  |
| 5 | Yadgir***** |  |

Districts 1-4 also had relatively higher MMR-DOC than other districts in 2014

* - Yadgir was part of Gulbarga district in 2007.The region was disadvantaged in all indicators in 2007 and 2013 (data not shown). ANC – Antenatal coverage; CANC – Complete Antenatal Coverage; SBA – Percentage of births attended by Skilled Birth Attendants. , BEmOC – Basic Emergency Obstetric Care; CEmOC – Comprehensive Emergency Obstetric Care. MMR-DOC – Maternal mortality due to Direct Obstetric Causes
